# Medium to long term follow up study of the efficacy of cessation of eye-rubbing to halt progression of keratoconus

**DOI:** 10.3389/fmed.2023.1152266

**Published:** 2023-05-24

**Authors:** Adrien Mazharian, Roxane Flamant, Sina Elahi, Christophe Panthier, Radhika Rampat, Damien Gatinel

**Affiliations:** Centre d’Explorations et de Recherche en Optique Visuelle (CEROV), Hôpital Fondation Rothschild, Paris, France

**Keywords:** eye-rubbing, keratoconus, cornea, ectasia, corneal topography, progression

## Abstract

**Purpose:**

To study the progression of keratoconus after cessation of eye rubbing with a minimum follow up of three-years.

**Design:**

Retrospective, monocentric, longitudinal cohort study of keratoconus patients with a minimum of 3 years follow-up.

**Participants:**

One hundred fifty three eyes of seventy-seven consecutive patients with keratoconus were included.

**Methods:**

Initial examination consisted of anterior and posterior segment evaluation using slit-lamp biomicroscopy. At the initial visit, patients were thoroughly informed of their pathology and instructed to stop rubbing their eyes. Eye rubbing cessation was assessed at all the follow-up visits at 6 months, 1 year, 2 years, 3 years, and yearly afterward. Corneal topography using the Pentacam® (Oculus®, Wetzlar, Germany) was used to obtain maximum and average anterior keratometry readings (Kmax and Kmean), as well as thinnest pachymetry (Pachymin, μm) in both eyes.

**Main outcome measures:**

The main outcomes measured were maximum keratometry (Kmax), mean keratometry (Kmean), and thinnest pachymetry (Pachymin) values at various time points to assess for keratoconus progression. Keratoconus progression was defined as a significant augmentation of Kmax (>1D), Kmean (>1D), or significant diminution of Pachymin (>5%) throughout the total follow-up duration.

**Results:**

One hundred fifty three eyes of seventy-seven patients (75.3% males) aged 26.4 years old, were followed for an average of 53 months. Over the course of the follow-up, there was no statistically significant variation of ∆Kmax (+0.04 ± 0.87; *p* = 0.34), ∆ Kmean (+0.30 ± 0.67; *p* = 0.27) nor ∆Pachymin (−4.36 ± 11.88; *p* = 0.64). Among the 26 of the 153 eyes which had at least one criterion of KC progression, 25 admitted continuing eye rubbing, or other at-risk behaviors.

**Conclusion:**

This study suggests that a significant proportion of keratoconus patients are likely to remain stable if close monitoring and strict ARB cessation are achieved, without the need for further intervention.

## 1. Introduction

Keratoconus (KC) is characterized by a progressive, asymmetric deformation of the cornea associated with central or paracentral corneal thinning, usually first diagnosed in adolescents or young adults. The condition results in ectasia, which can lead to significant visual impairment, sometimes severe enough to require corneal graft surgery (1).

The reported prevalence of eye-rubbing in KC patients is between 66% et 73% (2, 3). Although many different pathways such as biochemical, genetic, environmental, mechanical or multi-factorial origin have been investigated, the specific underlying cause of this condition is not fully understood (4).

Some hypothesized that repetitive, external, biomechanical stress can induce weakening and deformation of the cornea (5–7). Gatinel put forth the conjecture that KC is not a dystrophy of unknown genetics and biomolecular substratum, but rather a syndrome caused by eye rubbing, resulting in the progressive deformation and thinning of the corneal wall, the hallmarks of the disease (8, 9). If eye rubbing is an indispensable circumstance, then removing this mechanical stress could lead to stabilization of the induced corneal deformation (10, 11). This mechanical stress is often in the form of repetitive eye rubbing. Other at-risk behaviors (ARB) such as incorrect sleeping position (12–15) and applying overnight pressure on the cornea, may also play a role to some extent. While eye rubbing and allergy have recognized associations (16) with keratoconus, their precise causative relationship and contribution to disease progression is still debated. This study aimed to determine if eye rubbing, and other ARB cessation alone, may be effective in stabilizing keratoconus.

## 2. Materials and methods

### 2.1. Study design

This was a single-center retrospective cohort study of naïve keratoconus patients with a minimum of 3 years follow-up at the Rothschild Foundation, Paris, France.

The initial examination consisted of anterior and posterior slit lamp examination, corneal topography, maximum and mean keratometry (Kmax and Kmean, respectively), as well as thinnest pachymetry (Pachymin) (Pentacam®, Oculus®, Wetzlar, Germany). Objective refractive measures (Sphere and cylinder) recorded at initial visits were not included in the follow-up visits due to poor repeatability demonstrated in keratoconus patients (17). The study was conducted in accordance with the tenets of the 1964 Declaration of Helsinki. Informed consent was obtained from all the study subjects when anonymization was impossible.

### 2.2. Inclusion/exclusion criteria

Patients with keratoconus aged between 12 and 40 years old at the first visit were included based on the « Global consensus on keratoconus and corneal ectasia 2015 » criteria, defined as abnormal posterior ectasia, clinical non-inflammatory corneal thinning and abnormal corneal thickness distribution (18). Examinations were performed 3 days after soft contact lens and 2 weeks after rigid/scleral contact lens removal. Patients with consultations preceding their referrals often had missing or inconsistent data, clinical information, or underwent investigations using other imaging methods with poor comparability; the reference point (baseline) was defined as the first visit to our center. Patients with prior cross-linking, or surgical intervention were excluded as well as patients with factors leading to compulsive eye-rubbing (19) or poor compliance such as autism, Tourette’s syndrome, and Down Syndrome, a history of infectious corneal pathology, or presence of any inflammatory corneal condition, except for ocular allergies.

### 2.3. Outcome measures

The primary outcome measured was the absence of keratoconus progression, based on Wittig-Silva et al.’s criteria (20) of a significant increase in Kmax or Kmean (> 1.0D) or decrease in Pachymin (> 5%) over the course of the follow-up (defined as ∆Kmax, ∆Kmean, and ∆PachyMin). The standard criteria are variations over a one-year duration, but we restricted our study to the same criteria over the course of the entire follow-up duration to ensure that slowly-progressing eyes (i.e., <1D progression per year) do not falsely meet the absence of progression criteria.

### 2.4. Follow-up

Patients were examined and underwent topographic measurements at 6 months, 1 year, 2 years, 3 years, and yearly afterwards.

Most patients had multimodal imaging using several topographers, but the Pentacam® was chosen for this study because of its repeatability and reproducibility, as described in the literature (17, 21, 22). A subgroup of 100 anonymized patients consented to make their medical records, with clinical photographs and investigation reports available on the website: https://defeatkeratoconus.com (23).

### 2.5. Evaluation of at-risk behaviors

To evaluate the presence and extent of ARB, a detailed history was taken to establish the presence, frequency, and specific manner (frequency, intensity, dominant hand, time of day) of eye rubbing and other ARB, often aided by interrogation of family members and close relatives in patients suspected of anosognosia. This was repeated at each visit, and the importance of ARB cessation was reinforced. Patients describing ocular itch or discomfort were prescribed topical antihistamines and/or ocular lubricants. Patients with potentially damaging sleeping positions (prone or side-sleeping position with ocular compression) were systematically offered a rigid eye shields as nighttime protection and encouraged to modify their sleeping position (12–15).

Only repeated and daily eye-rubbing with the testimony of relatives was considered pathological. We have collected all of these testimonials, including making case reports with photos by putting them on the following site: https://defeatkeratoconus.com/ (23).

We have yet to find another way to characterize this behavior, which remains mainly based on the patient’s declaration.

Sleeping with either eyes dug in the pillow or nocturnal eye-rubbing was considered pathological. Sleeping on the back or the opposite side was not counted as an ARB. Therefore, a patient reporting a predominant right-sided sleeping position had only the right eye included in the ARB group.

### 2.6. Statistical analysis

Results were reported as mean ± standard deviation (SD). ∆Kmax, ∆Kmean, ∆Pachymin were defined as the difference between the values at the latest visit and the baseline.

A statistically significant difference between means and between timepoints was determined using paired or unpaired Student *t*-test, as well as a categorical pairwise comparison using Tukey’s method. value of ps <0.05 were considered statistically significant. All statistical analyses were performed using Stata software (v14.0 StataCorp, TX, United States).

## 3. Results

### 3.1. Baseline patient characteristics

**Figure 1:**
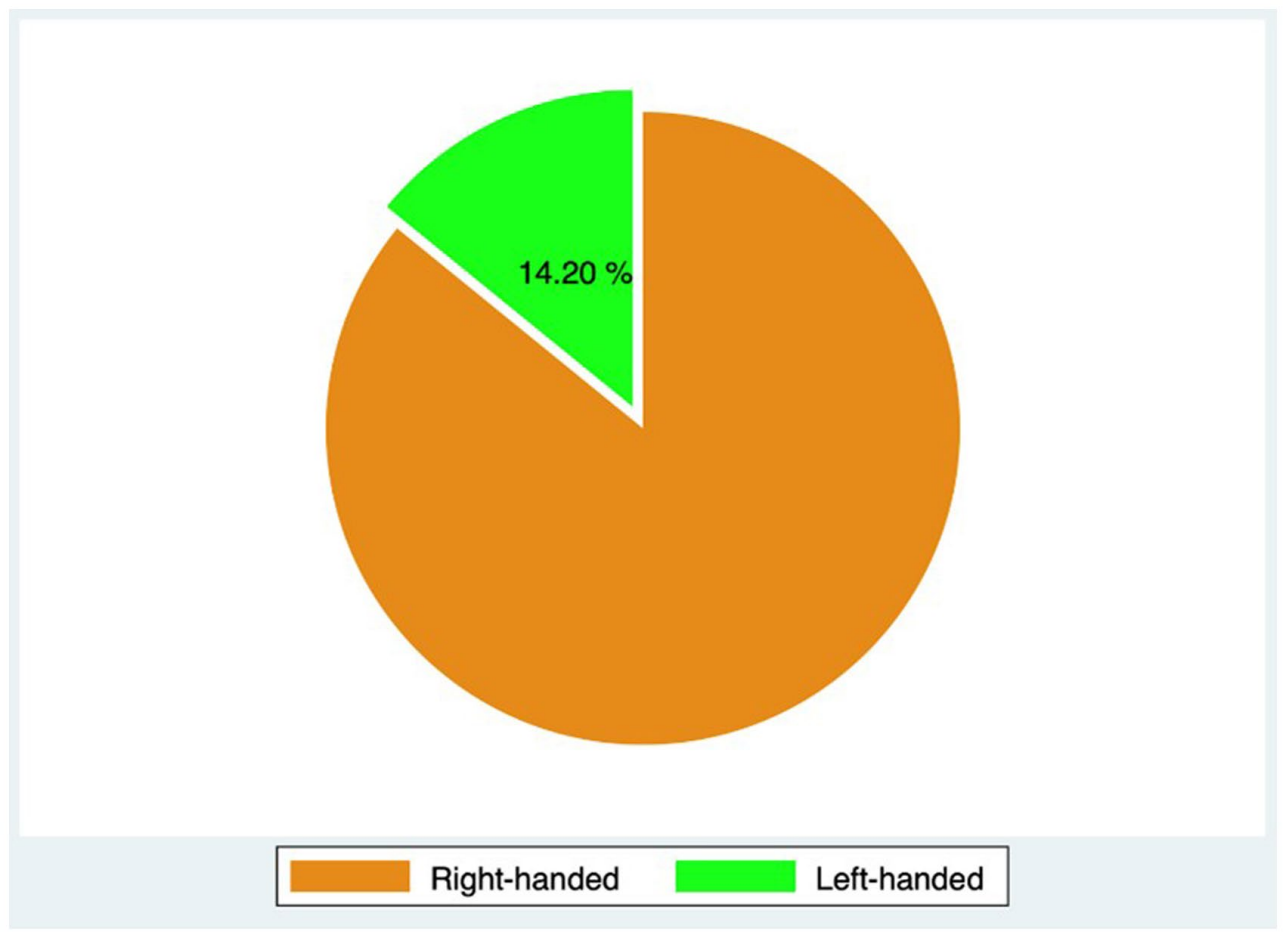
Distribution of dominant hand in the cohort.

Out of 396 patients diagnosed with keratoconus during the studied period, 176 met the inclusion criteria, and 77 completed the 3-year follow-up. The study included 153 eyes (77 patients, 75.3% males) with keratoconus over a follow-up of 53 ± 20.8 months. The average age was 26.4 years (range 12–40 years). Table 1 displays demographic and baseline characteristics. Figure 1 shows the distribution of dominant hand in the studied population. Table 2 displays demographic and baseline characteristics in each studied sub-group:: absence of At-Risk Behavior (ARB) or Recurrent Eye-Rubbing (RER), presence of ARB only, presence of RER only and presence of both ARB and RER.

**Table 1:** Baseline characteristics of patients.

	Value	SD or %
Total of patients	77	0
Number of KC eyes	153	0
Age (years)	26.4	± 7.3
Male, *n* (patients)	58/77	75.32%
Follow-up (months)	53.0	±20.8
Kmax	51.6	± 6.1
Kmean	45.5	± 3.2
Pachymin (μm)	479.3	± 41.9

KC, Keratoconus; Kmax, Maximal keratometry reading; Kmean, Mean keratometry reading; PachyMin, Minimal corneal thickness measurement; SD, Standard Deviation.

**Table 2:** Baseline characteristics of patients in each sub-group.

	No ARB/RER	RER only	ARB only	ARB & RER	*p*-value
Total of patients	44	15	18	10	
Number of KC eyes	80	28	28	17	
Age (*years*)	26.5	26.7	27.4	30.1	0.7
Follow-up (*months*)	46.6	41.0	53.0*	55.1*	*<0.01*
Kmax	52.2	50.3	51.0	50.5	0.44
Kmean	46.1	45.2	44.9	44.1	0.06
Pachymin (μm)	475.7	475.0	496.9	484.5	0.20

ARB, At-Risk Behavior; RER, Recurrent Eye-Rubbing; KC, Keratoconus; Kmax, Maximal keratometry reading, Kmean, Mean keratometry reading; PachyMin, Minimal corneal thickness measurement, *Indicates statistical significance in this sub-group (*p* < 0.05).

### 3.2. Keratometry measures (Kmax and Kmean)

The variation in keratometry at various time points is presented in Tables 3, 4. There is no statistically significant difference between ∆Kmax means at all time points, as displayed on Table 3. Similar results were observed for ∆Km with almost no significant difference at all time points except between year 2 and year 3 (0.09 ± 0.86 vs. 0.56 ± 1.97, *p* = 0.03) (Table 4). The variation in keratometry at various time point has also been analyzed in 4 sub-groups: absence of At-Risk Behavior (ARB) or Recurrent Eye-Rubbing (RER), presence of ARB only, presence of RER only and presence of both ARB and RER. They are summarized in Tables 5, 6. There is a statistically significant difference of Kmax variation in the “RER only” sub-group from month from year 2 until end of follow-up for Kmax (+1.48, *p* = 0.04, +2.32, *p* < 0.01, +4.01, *p* < 0.01 and + 1.60, *p* < 0.01) (Table 5) and year 3 until end of follow-up for Kmean (+1.14, *p* < 0.01, +3.58, *p* < 0.01 and + 1.17, *p* < 0.01) (Table 6). Other sub-groups show no significant variation in both Kmax and Kmean during follow-up, except for “ARB and RER” sub-group regarding Kmean criteria at more than 48 months follow-up (+0.60, *p* = 0.02) (Tables 5, 6).

**Table 3:** Maximal Keratometry^1^ variations at different timepoints.

	Mean	SD	N	*p* ^‡^
Baseline	0	0	153	
Δ M6	0.01	1.01	78	0.91
Δ M12	−0.16	0.72	107	0.23
Δ M24	−0.05	1.81	90	0.56
Δ M36	0.57	2.76	116	0.07
Δ M48	0.46	3.18	72	0.83
Δ > M48	0.04	0.87	51	0.34

M, Months; SD, Standard Deviation. ^‡^Unpaired Student’s *t*-test comparing the statistically significant difference from the previous timepoint. *Indicates statistical significance (*p* < 0.05). ^1^Stand for Kmax.

**Table 4:** Mean Keratometry^2^ variations at different timepoints.

	Mean	SD	N	*p* value^‡^
Baseline	0	0	153	
Δ M6	0.04	0.76	86	0.52
Δ M12	−0.04	0.48	110	0.39
Δ M24	0.09	0.82	90	0.18
Δ M36	0.56	1.97	120	0.03*
Δ M48	0.66	2.48	84	0.75
Δ > M48	0.30	0.67	61	0.27

M, Months; SD, Standard Deviation. ^‡^Unpaired Student’s *t*-test comparing the statistically significant difference from the previous timepoint. *Indicates statistical significance (*p* < 0.05). ^2^Stand for Kmean.

**Table 5:** Maximal Keratometry^1^ variations at different timepoints for each sub-group: no ARB nor RER, ARB only, RER only and both ARB and RER.

∆KMAX	No ARB/RER	RER only	ARB only	ARB & RER	***N***	*p* value^***‡***^
Baseline	0	0	0	0		
Δ M6	−0.17	0.26	−0.18	−0.89	78	0.07
Δ M12	−0.23	−0.34	0.01	−0.20	107	0.68
Δ M24	−0.53	1.48*	−0.26	0.04	90	0.04
Δ M36	0.07	2.32*	0.38	−0.06	115	<0.01
Δ M48	−0.18	4.01*	−0.03	−0.54	72	<0.01
Δ > M48	−0.31	1.6*	−0.02	1.22	52	<0.01

ARB, At-Risk Behavior, RER, Recurrent Eye-Rubbing, M, Months, ^‡^Unpaired Student’s *t*-test comparing the statistically significant difference from the previous timepoint, *Indicates statistical significance in this sub-group (*p* < 0.05). ^1^Stand for Kmax.

**Table 6:** Mean Keratometry^2^ variations at different timepoints for each sub-group: no ARB nor RER, ARB only, RER only and both ARB and RER.

∆KM	No ARB/RER	RER only	ARB only	ARB & RER	*N*	*p* value^**‡**^
Baseline	0	0	0	0		
Δ M6	−0.07	−0.10	0.08	−0.10	86	0.39
Δ M12	−0.04	−0.01	−0.10	0.00	110	0.96
Δ M24	−0.12	0.76	0.23	0.17	90	0.07
Δ M36	0.34	1.14*	0.39	0.16	119	<0.01
Δ M48	0.66	3.58*	0.31	−0.13	84	<0.01
Δ > M48	0.05	1.17*	0.07	0.60*	62	0.02

ARB, At-Risk Behavior, RER, Recurrent Eye-Rubbing, M, Months, ^‡^Unpaired Student’s *t*-test comparing the statistically significant difference from the previous timepoint, *Indicates statistical significance in this sub-group (*p* < 0.05). ^2^Stand for Kmean.

### 3.3. Minimal pachymetry

Variations in Pachymin are reported throughout Table 7 and show a statistically significant difference between ∆Pachymin at year 2 and year 3 (−0.24 ± 10.26 vs. –4.38 ± 14.18, p = 0.02). The variation in Pachymin at various time point has also been analyzed in 4 sub-groups: no At-Risk Behavior (ARB) nor Recurrent Eye-Rubbing (RER), ARB only, RER only and both ARB and RER. They are summarized in Table 8. “Only RER” sub-group shows statistically significant difference in Pachymin variation from year 2 to year 4 (−7.33, *p* = 0.01, −15.34, *p* = 0.03 and-22.46, *p* < 0.01). “ARB and RER” sub-group presents significant Pachymin variation at year 2 and 4 (+5.38, *p* = 0.01 and + 6.20, *p* < 0.01). As this variation is positive, it cannot be associated with keratoconus progression. Other sub-groups display no statistically significant difference in Pachymin (Table 8).

**Table 7:** Minimal pachymetry outcomes in keratoconus patients.

	Mean	SD	*N*	*p* ^‡^
Baseline	0	0	153	
Δ M 6	−0.42	9.563	86	0.59
Δ M 12	−0.81	8.41	110	0.76
Δ M 24	−0.24	10.26	90	0.67
Δ M 36	−4.38	14.18	120	0.02*
Δ M 48	−3.25	15.52	84	0.59
Δ > M48	−4.36	11.88	61	0.64

M, Months, SD, Standard Deviation. ^‡^Unpaired Student’s *t*-test comparing the statistically significant difference from the previous timepoint. *Indicates statistical significance (*p* < 0.05).

**Table 8:** Minimal pachymetry outcomes in keratoconus patients for each sub-group: no ARB nor RER, ARB only, RER only and both ARB and RER.

∆PachyMin (μm)	No ARB/RER	RER only	ARB only	ARB & RER	*N*	*p*-value
Baseline	0	0	0	0		
Δ M 6	1.51	−1.58	−4.41	7.50	86	0.07
Δ M 12	0.34	−2.64	−1.72	−2.00	110	0.32
Δ M 24	−0.34	−7.33*	2.27	5.38*	90	0.01
Δ M 36	0.03	−15.34*	−4.82	−4.38	119	0.03
Δ M 48	0.44	−22.46*	−2.23	6.20*	84	<0.01
Δ > M48	−4.65	−0.25	0.67	−3.33	62	0.76

ARB, At-Risk Behavior, RER, Recurrent Eye-Rubbing, M, Months, ^‡^Unpaired Student’s *t*-test comparing the statistically significant difference from the previous timepoint, *Indicates statistical significance in this sub-group (*P* < 0.05).

### 3.4. Progression analysis

#### 3.4.1. Primary outcome: Kmax ≥ 1D

Out of the 153 studied eyes, 21 (13.73%) showed progression according to the ∆Kmax criteria, 20 of which (95.23%) admitted that they could not stop their ARB.

**Figure 2:**
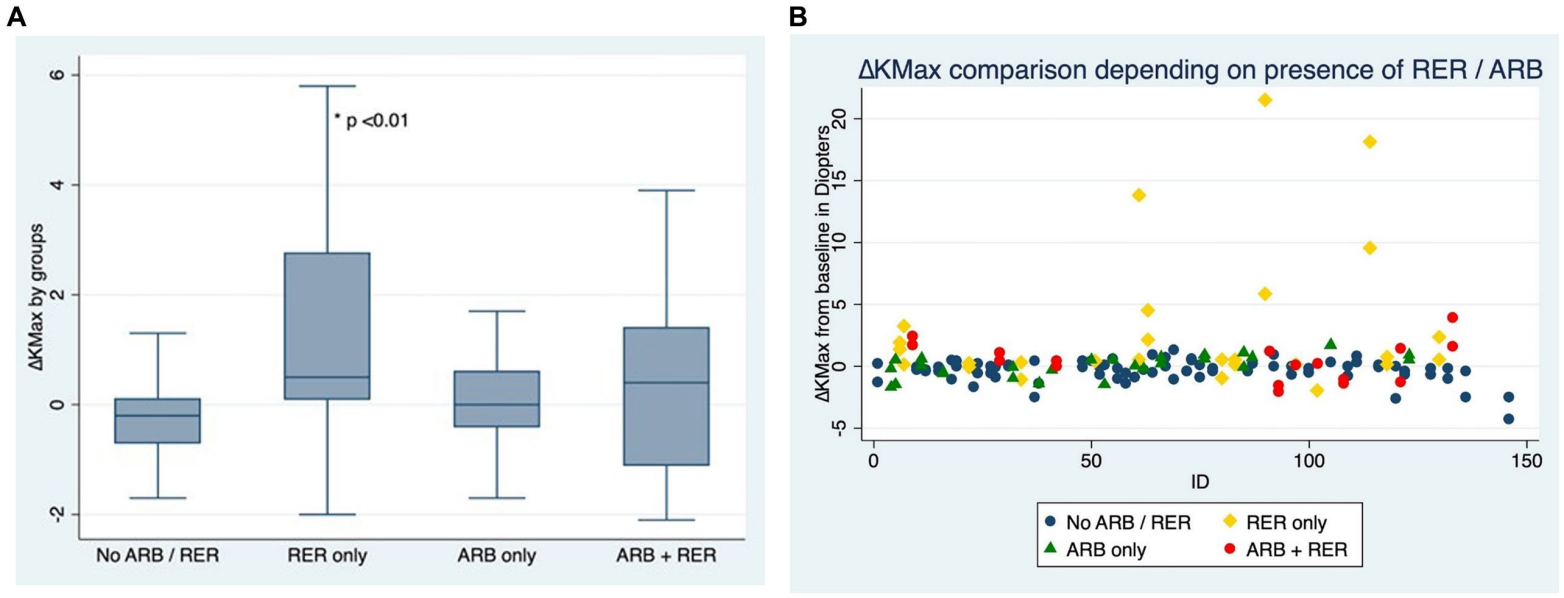
**(A)** Comparison of Kmax variation for each eye, depending on presence or absence of at-risk behaviors (ARB) and recurrent eye-rubbing (RER) - Box and whiskers for each sub-group. **(B)** Comparison of Kmax variation for each eye, depending on presence or absence of at-risk behaviors (ARB) and recurrent eye-rubbing (RER) - “Scattered-Dot” Plots Graph for each sub-group.

Figures 2A,B compare ∆Kmax at the end of the follow-up between patients who have kept both ARB and RER, one of each and patients who have managed to stop.

#### 3.4.2. Secondary outcome: Kmean

Out of the 153 eyes, 16 (10.46%) showed progression according to the ∆Kmean secondary criteria, all of which (100%) failed to stop ARB over the course of the follow-up.

**Figure 3:**
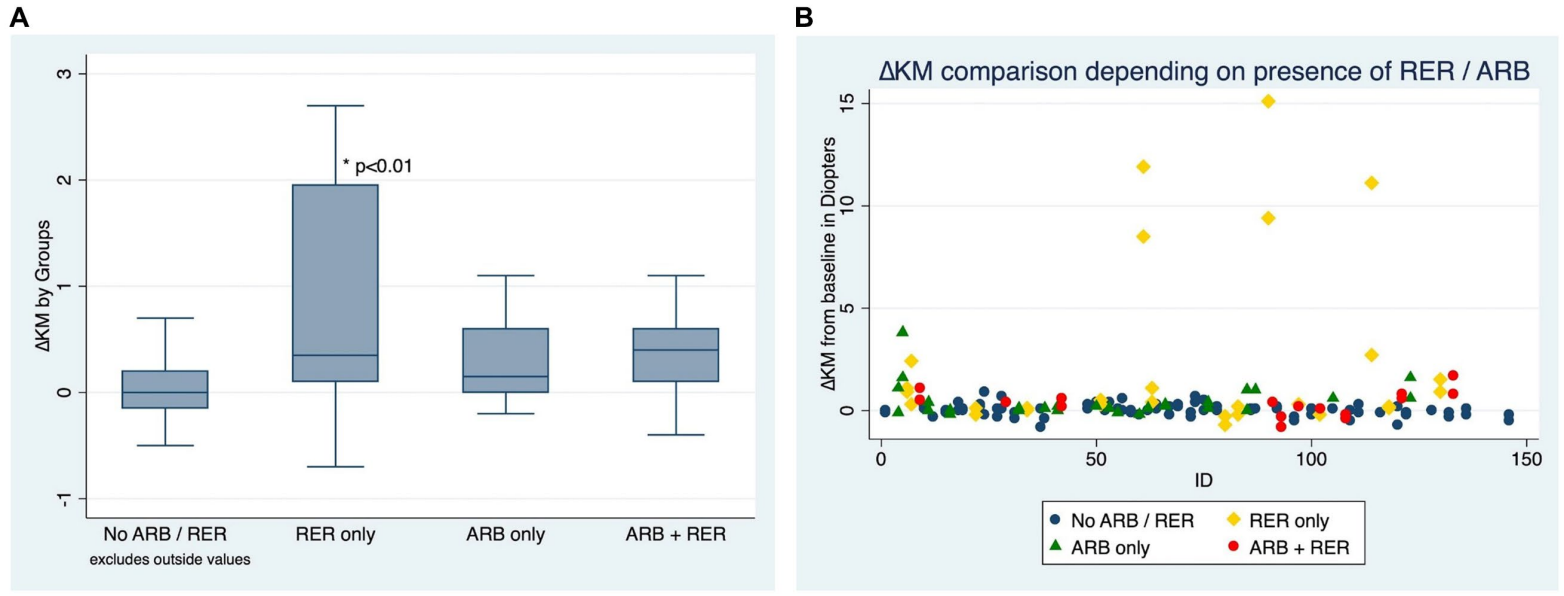
**(A)** Comparison of Kmean variation for each eye, depending on presence or absence of at-risk behaviors (ARB) and recurrent eye-rubbing (RER) - Box and whiskers for each sub-group. **(B)** Comparison of Kmean variation for each eye, depending on presence or absence of at-risk behaviors (ARB) andrecurrent eye-rubbing (RER) - “Scattered-Dot” Plots Graph for each sub-group.

Figures 3A,B compare ∆Km at the end of the follow-up between patients who have kept both ARB and RER, one of each and patients who have managed to stop.

#### 3.4.3. Secondary outcome: PachyMin

Out of the 153 eyes, 7 (4.58%) showed progression according to the ∆PachyMin secondary criteria, all of which (100%) failed to stop ARB during the follow-up.

**Figure 4:**
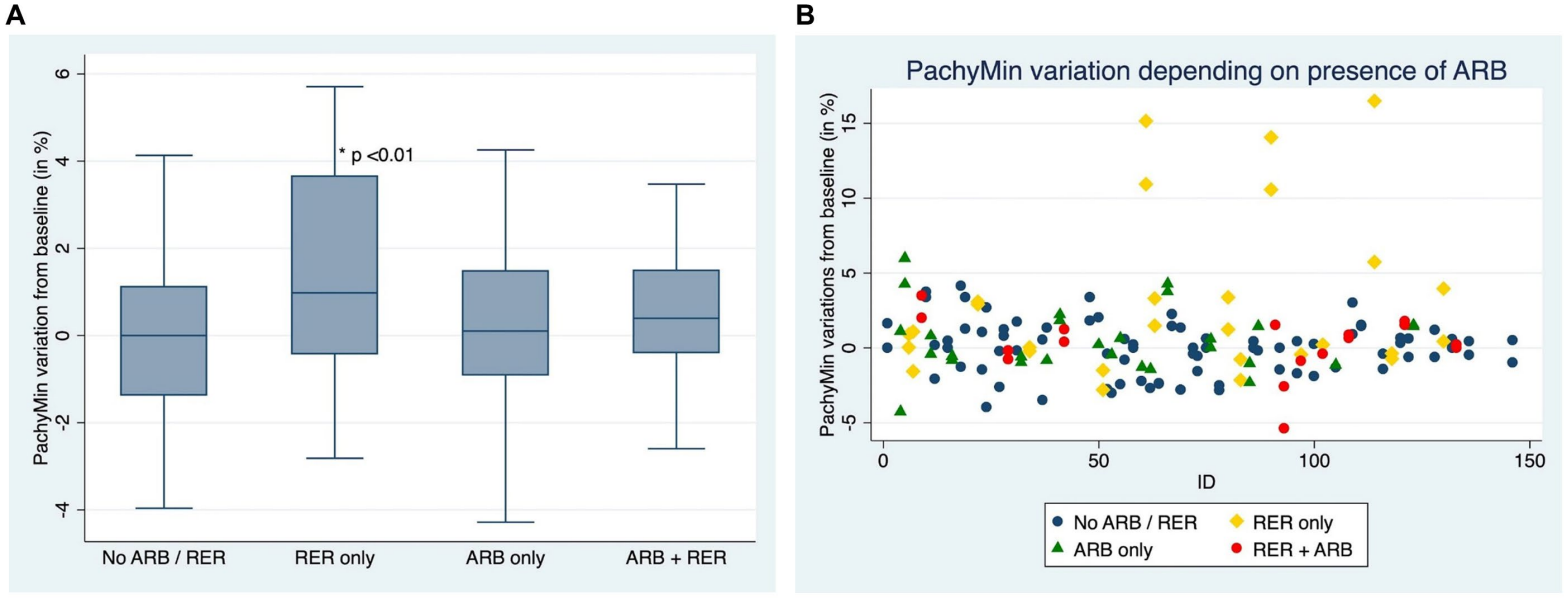
**(A)** Comparison of Pachymin variation for each eye, depending on presence or absence of at-risk behaviors (ARB) and recurrent eye-rubbing (RER) - Box and whiskers for each sub-group. **(B)** Comparison of Pachymin variation for each eye, depending on presence or absence of at-risk behaviors (ARB) and recurrent eye-rubbing (RER) - “Scattered-Dot” Plots Graph for each sub-group.

Figures 4A,B compare ∆Pachymin at the end of the follow-up between patients who have kept both ARB and RER, one of each and patients who have managed to stop.

Out of 153 eyes, 26 had at least one progression criterion, and among these, 23 continued ARB.

3/26 patients had disease progression despite positively denying any form of ARB. When comparing progressive and non-progressive populations, there was no statistically significant difference in terms of baseline age, Kmax, Kmean or pachymin as shown in Table 9. Table 10 details the percentage of progression for each positive criteria, in all pre-cited sub-groups. The “RER only” sub-group shows the highest progression rate for each criteria (35.71 and 21.43% for Kmean and Pachymin respectively), except for Kmax>1D: “RER and ARB” sub-group ranks first in this category (41.18% vs. 39.29% for “RER only) as displayed in Table 10.

**Table 9:** Characteristics of patients with and without progression.

	General	Progressing	Non progressing	*p*-value
Total of patients	77			
Number of KC eyes	153	26 (16.99%)	127 (83.01%)	
Age (years)	26.4	24.83	26.70	0.24
Male (percentage of patients)	75.82%	69.23%	77.13%	0.39
Follow-up (months)	53.04	57.74	52.08	0.21
Kmax	51.6	53.47	51.16	0.08
Kmean	45.5	46.02	45.43	0.39
Pachymin (μm)	479.3	474.35	480.32	0.51

KC, Keratoconus; Kmax, Maximal keratometry reading, Kmean, Mean keratometry reading; PachyMin, Minimal corneal thickness measurement.

**Table 10:** Percentage of progression according to positive criteria in each sub-group.

Progression	KMAX (>1D)	KM (>1D)	PACHYMIN (>5%)
No ARB/RER	1/80 (1.25%)	0/80 (0%)	0/80 (0%)
RER only	11/28 (39.29%)	10/28 (35.71%)	6/28 (21.43%)
ARB only	2/28 (7.14%)	4/28 (14.29%)	1/28 (3.57%)
ARB & RER	7/17 (41.18%)	2/17 (11.76%)	0/17 (0%)

ARB, At-Risk Behavior; RER, Recurrent Eye-Rubbing; Kmax, Maximal keratometry reading; Kmean, Mean keratometry reading; PachyMin, Minimal corneal thickness measurement.

## 4. Discussion

This retrospective study describes the relationship between ARB (especially eye rubbing) and KC progression, and the possibility that solely preventing the corneas to suffer repetitive mechanical stress, may be sufficient for long-term disease stabilization (or delay in progression), thereby avoiding the risks of potentially unrequired treatments. Multiple studies have reported a higher prevalence of eye-rubbing in keratoconus patients compared to the normal population (2, 4, 6, 24–29), and the implication of eye-rubbing in development and progression of keratoconus is well-described in the literature (30–33). Eye-rubbing has been shown to be the most significant cause of keratoconus in multivariate analysis (34). However, whether eye-rubbing is merely a risk-factor, or rather a sine-qua-non condition for the disease to appear and progress, is a question that remains yet debated (8, 9, 35).

The results of this study show disease progression (according to any of the three defined criteria) in 16.9% (26/153) of the eyes. Almost all were explained by the self-admitted persistence of ARB. Only 1 of these (0.65%) showed progression in the absence of ARB. Careful and thorough evaluation of the files and clinical data did not permit to identify ARBs or risk factors explaining the progression. Due to the nature of the ARBs, which are most often subconscious and can even occur overnight while unconscious, we believe that there is a probability of anosognosia. Objective confirmation of this hypothesis is unlikely to be proven until technology makes objective continuous monitoring of ARB possible.

*Ferdi* et al. systematic review and meta-analysis (11,529 eyes) (36) studying keratoconus’ natural progression found a significant Kmax progression of 0.7D at 12 months follow-up with significant variation depending on age and ethnicity. A significant Kmean progression was measured at 0.4D during the same period. Their meta-analysis model predicted a gradual reduction in the progression of Kmax and Kmean over the years. When comparing a control-group with a cross-linking group, Wittig-Silva et al. (20) reported a mean Kmax variation of 1.20 vs. –0.72D at 12 months and 1.75D vs. –1.03D at 36 months, respectively. Minimal pachymetry reduction at 36 months was 17.01 vs. 19.52 μm in the control and cross-linking groups, respectively.

Finally, a systematic review and meta-analysis of two techniques of crosslinking (transepithelial versus epithelium-off) (37) reported a KMax variation of-0.06D at 12 months in the transepithelial cross linking group (440 eyes) compared to-0.54D in the epithelium-off group (486 eyes). Kmean progression was measured at-0.62D and-1.48D at 12 months and progression at 12 months was 7 and 2% in the trans-epithelial and epithelium-off groups, respectively. Significant complications such as corneal melt, persistent corneal ulcer, or visually significant stromal haze were seen in 2 and 4% of eyes in the trans-epithelial and epithelium-off groups, respectively.

Our patients showed a mean Kmax progression of-0.16D and + 0.57D at 12 et 36 months, respectively, which is better than the natural progression reported by *Ferdi* et al. meta-analysis (36) and Wittig-Silva et al. control-group (20). We report a Kmean variation of-0.16D and + 0.53D at 12 et 36 months, respectively, which outperforms Ferdi et al. meta-analysis (36) again. Regarding the pachymin criteria, we demonstrate a loss of 0.42 μm and 4.4 μm at 12 and 36 months, respectively, which surpasses both Ferdi et al. (36) and Wittig-Silva et al. (20) groups.

Our cohort thus demonstrates fewer variation in all progression criteria compared to natural keratoconus progression as defined by Ferdi et al. large meta-analysis (36) and Wittig-Silva et al. control-group (20).

Regarding crosslinking data, both Wittig-Silva et al. (20) and Nath et al. (37) reported a significant Kmax flattening which seems to persist up to 36 months follow-up and outperforms our results. However, our keratoconus progression rate (1.96% in the absence of reported eye-rubbing or ARB at 53 months mean follow-up) equals if not supersedes Nath et al. (37) two cross-linking groups (2 and 7% at 12 months follow-up). One could discuss that cross-linked populations usually present more aggressive forms of keratoconus, resulting in a selection bias in those cohorts. But one should remember that our patients were all showing progression when referred to our department. On the other hand, one should also consider the significant complications rate observed by *Nath* et al. systematic review and meta-analysis (37), compared to their absence in our conservative approach.

In conclusion, our conservative treatment performs well with regards to keratoconus progression rate, in comparison to both its natural evolution and cross-linked populations. It can be described as a safe and efficient first-step treatment of keratoconus, which resolves most keratoconus progression cases. More invasive procedures, such as cross-linking can be indicated as second-step treatment if progression persists despite good compliance to recommendations.

When asked about performing anticipated cross-linking in pediatric patients, in absence of progression proof, we think that recent literature shows controversy in terms of cross-linking efficacy and safety, especially in pediatric populations. On one hand, Ferdi et al. (36) meta-analysis predicts an annual 1.5D Kmax progression in under 17 years old patients and McAnena et al. (38) meta-analysis and systematic review recommends cross-linking in pediatric keratoconus cases. On the other hand, Or et al. (39) five year follow-up suggests that there a no emergency to perform cross-linking in underaged patients in absence of progression proof, and Achiron et al. (40) meta-analysis observes a rough 10% keratoconus progression risk after cross-linking in this same population. As therapists, we feel that performing a procedure carrying significant risks without proof of progression is aggressive.

Our approach would rather favor patient education to their chronic disease, removal of causative factors and close monitoring of high-risk populations such as the pediatric one. If progression is observed, further treatment such as cross-linking is more promptly and aggressively suggested, according to McAnena et al. (38) conclusions.

Regarding data, our cohort contains some young patients. Twenty-six patients were younger than 18 at diagnosis and were included in this study. When comparing progression criteria in this subgroup to the older patients, there was progression in 5/26 (19.23%) compared to 16/127 (12.60%) (*p* = 0.37). Of the 5 young patients progressing, 4 admitted continuing daily eye rubbing despite the recommendations.

We hypothesize that young people are less compliant with the recommendations, probably because of poor understanding of the possible seriousness of their condition. We believe that if these patients developed keratoconus at an early age, it is because of more aggressive eye-rubbing habits, which are more difficult to give up.

Conversely, allergic conjunctivitis was more often reported in those patients’ files than in older ones. Topical treatment was key to helping them eradicate their eye-rubbing habits, and thus keratoconus progression.

Finally, there is also known physiologic evidence that Maillard reaction enhances cornea rigidity in aging corneas (41). As younger patients do not benefit from this protective factor, we believe that shear-stress caused by eye-rubbing tends to be more impactful on their corneas, leading to more progress.

Mechanical models have shown that keratoconus progresses under environmental stresses, but only when there is an initial defect, especially a thinning defect such as that induced by continual eye rubbing (42). McMonnies (6) extensively reviewed the possible mechanisms for the association between chronic habitual eye rubbing and the development of keratoconus. He described several potential factors, including temperature increase from eye rubbing (2, 43–45), leading to increased activity of inflammatory mediators and enzymes; hydrostatic pressure increases combined with enzyme activation “tenderizing” the cornea; reduction of corneal shear strength; reduction of proteoglycan viscosity and displacement of proteoglycans from the corneal apex; and induction of keratocyte apoptosis from eye rubbing. It has been shown that healthy patients who rub their eyes mainly use the pulps of their fingers, producing a weak force equivalent to 0.45 cm kg/2.54^2^, while keratoconus patients tend to use their knuckles, along with more frequent and prolonged rubbing, producing a force greater than 4.5 cm kg/2.54^2^ (46). Indeed there are many anecdotal reports in the literature which support a mechanical hypothesis, with reports of keratoconus associated with repeated and vigorous eye-rubbing alone in patients without a family history of KC (33, 47–50).

In addition, the mechanical hypothesis is consistent with the well-recognized association between allergy and keratoconus. In certain countries such as Israel (51), Lebanon (52), and Saudi Arabia (53), the association with allergy is even stronger. Patients with ocular allergy are more often subject to itch, therefore more likely to rub their eyes and induce further progression of keratoconus. Keratoconus has been shown to be more likely to progress in patients with ocular allergy (53, 54).

It is increasingly recognized that the development and progression of keratoconus is highly likely in the presence of external triggers, particularly repetitive vigorous corneal trauma such as eye-rubbing, compression of the globe during sleep (15), heat (43) and induced inflammation, atopy, and allergy, which may be or may not be associated with a coexisting genetic predisposition which renders the cornea more susceptible to trauma. This is then responsible for a cascade of biochemical events culminating in the development of keratoconus. Notably, only 6 patients in our study had a family history of keratoconus, while all patients admitted vigorous eye-rubbing, and indeed a specific genetic factor for keratoconus has proven difficult to identify. Although certain familial cases and genetic studies favor a genetic origin (55), no specific gene mutation has been clearly identified, and discordance for keratoconus in monozygotic twins have been reported (56).

One of the alarming issues with studies pertaining to keratoconus is the lack of consensus regarding the definition of disease progression, which often relies on the interpretation of multiple parameters such as subjective refraction, uncorrected and best-corrected visual acuity, and corneal topographies. Despite its important flaw of limiting the disease to just a focal corneal measurement, an increase in the maximum keratometry (≥1 D) remains the most frequently reported index of disease progression (18, 21, 22). However, a rising trend among corneal specialists suggests several criteria, especially non-focal ones, may prove more reliable (57).

We acknowledge that our study has limitations. Although patients are encouraged to maintain their follow-up, significant loss to follow-up occurred in the initial population. This is likely explained by the nature of the center, a specialized tertiary center in a capital city that receives many referrals from patients with long commutes who may prefer local care after the initial first specialized visits. This might explain the statistically significant difference in ∆Kmean and ∆PachyMin at one specific timepoint (year 3) with a loss of significance at later time points. The absence of further treatment escalation, such as an intervention date, in addition to the seemingly simplistic therapeutic approach proposed (in the eyes of the patients), may also have undermined the importance of regular follow-up in a population composed of young and active individuals with professional obligations. It has been observed that cross-linking studies are susceptible to loss of follow-up of between 6 and 15% depending on the duration of study (58). The loss of follow-up may have biased the results of this study by excluding patients with relatively mild to moderate forms of stabilized keratoconus. In our experience, the present cohort illustrates a more pessimistic view of keratoconus progression than what one actually sees in daily practice.

The design of our study, which is based on retrospective self-reported behaviors, inevitably leads to recall bias. However, we did not find other better ways to assess and monitor patients’ behavior. Progress in objective hand gesture detection and eye rubbing through monitoring devices such as smartwatches, as developed during the covid-pandemic to detect eye-or nose-touching (59), might be a solution in the future.

Meanwhile, eye-rubbing and its associated behaviors are, from our experience, often unconscious habits, and patients require time and awareness to detect and eradicate them. We take time with them during medical consultation, including listening to the testimonies of their relatives with them. We are aware of the risk that eye rubbing could represent for them, as well as being more careful not to perform this gesture. They are then often underestimated by the patient at their first appointment and later estimated more realistically either by the patient or their close ones. They often admit to having underestimated their unconscious habit at their second appointment.

The baseline was defined as the first consultation at the center, which ignores previous investigations and consultations in other centers and makes evaluating previous progression rates impossible. All patients were progressing at the time of referral to our tertiary center. Patients were referred either after initial diagnosis due to visual loss or because of progression observed by their referring ophthalmologist. We acknowledge that this fact, combined with the study’s monocentric and retrospective nature, may lead to a partial selection bias which may hinder the generalization of our results to other populations.

However, the inclusion criteria and the outcome measures are comparable to non-pediatric studies evaluating long-term efficacy and tolerance after corneal cross-linking (CXL) (20, 57, 60).

The robust methods in our study and the use of objective judgment criteria and multimodal imaging with good repeatability also limit this potential bias. As the outcome measures were objective and quantitative, with measurements taken by independent operators, the influence on results is limited.

By further analyzing the patient’s profile of progressing eyes, eye rubbing continuation despite recommendations predominated. However, careful follow-up must be maintained for all patients, and treatment escalation might be offered if progression is detected despite good compliance with recommendations.

Larger studies and longer follow-ups are needed to support our findings. While the relationship between mechanical trauma from eye rubbing and disease progression in keratoconus remains incompletely understood, based on these results, we believe that cessation of eye-rubbing is an important first step toward halting the progression of this condition in keratoconus patients. Our study demonstrates stable Kmax, Kmean, and Pachymin following cessation of eye-rubbing over a 3-year follow-up period. This highlights the importance of screening for eye-rubbing during patient evaluations, and our results suggest that definitive cessation of eye rubbing alone (albeit difficult) can be successful in limiting progression or achieving stabilization of keratoconus in the long-term in almost all eyes.

## Data availability statement

The raw data supporting the conclusions of this article will be made available by the authors, without undue reservation.

## Ethics statement

The studies involving human participants were reviewed and approved by Ethic committee of Fondation Ophtalmologique Adolphe de Rothschild, Paris, France. Written informed consent for participation was not required for this study in accordance with the national legislation and the institutional requirements.

## Author contributions

AM wrote the core of this manuscript and initiated the database of the study. RF collected all the data and participated to the writing of the manuscript. SE did the statistical analysis of the data and participated to the writing of the manuscript. CP and RR reviewed the manuscript and conducted data analysis. DG reviewed the manuscript and was the instigator of both the project and the hypothesis developed in this manuscript. All authors contributed to the article and approved the submitted version.

## Conflict of interest

The authors declare that the research was conducted in the absence of any commercial or financial relationships that could be construed as a potential conflict of interest.

## Publisher’s note

All claims expressed in this article are solely those of the authors and do not necessarily represent those of their affiliated organizations, or those of the publisher, the editors and the reviewers. Any product that may be evaluated in this article, or claim that may be made by its manufacturer, is not guaranteed or endorsed by the publisher.

## References

[1] Rabinowitz YS. Keratoconus. Surv Ophthalmol. (1998) 42:297–9. doi: 10.1016/S0039-6257(97)00119-7 9493273

[2] McMonnies CW. Eye rubbing type and prevalence including contact lens « removal-relief » rubbing. Clin Exp Optom juill. (2016) 99:366–2. doi: 10.1111/cxo.12343, PMID: 27306478

[3] Moran S Gomez L Zuber K Gatinel D. A case-control study of keratoconus risk factors. Cornea. juin. (2020) 39:697–1. doi: 10.1097/ICO.0000000000002283 32040008

[4] Sugar J Macsai MS. What causes keratoconus? Cornea juin. (2012) 31:716–9. doi: 10.1097/ICO.0b013e31823f8c72 22406940

[5] Norouzpour A Mehdizadeh A. A novel insight into keratoconus: mechanical fatigue of the cornea. Med Hypothesis Discov Innov Ophthalmol J. (2012) 1:14–7. PMC3939737 24600612

[6] McMonnies CW. Mechanisms of rubbing-related corneal trauma in keratoconus. Cornea juill. (2009) 28:607–5. doi: 10.1097/ICO.0b013e318198384f 19512912

[7] McMonnies CW Schief WK. Biomechanically coupled curvature transfer in normal and keratoconus corneal collagen. Eye Contact Lens janv. (2006) 32:51–62. doi: 10.1097/01.icl.0000183177.22734.f3, PMID: 16415695

[8] Gatinel D. Eye rubbing, a sine qua non for keratoconus? Int J Keratoconus Ectatic Corneal Dis. (2016) 5:6–12. doi: 10.5005/jp-journals-10025-1114 PMC5627975 28989906

[9] Gatinel D. Challenging the “no rub, no cone” keratoconus conjecture. Int J Keratoconus Ectatic Corneal Dis. (2018) 7:66–81. doi: 10.5005/jp-journals-10025-1161

[10] McMonnies CW. Management of chronic habits of abnormal eye rubbing. Contact Lens Anterior Eye J Br Contact Lens Assoc avr. (2008) 31:95–2. doi: 10.1016/j.clae.2007.07.008 18356094

[11] McMonnies CW. Behaviour modification in the management of chronic habits of abnormal eye rubbing. Contact Lens Anterior Eye J Br Contact Lens Assoc. avr. (2009) 32:55–63. doi: 10.1016/j.clae.2008.11.001, PMID: 19188087

[12] de Azevedo MO Gonçalves MC Gatinel D. The role of environment in the pathogenesis of keratoconus. Curr Opin Ophthalmol. (2021) 32:379–4. doi: 10.1097/ICU.0000000000000764 33966012

[13] Gatinel D Galvis V Tello A Niño CA Rey JJ Camacho PA. Obstructive sleep Apnea-hypopnea syndrome and keratoconus: an epiphenomenon related to sleep position? Cornea avr. (2020) 39:e11–2. doi: 10.1097/ICO.0000000000002219, PMID: 31764281

[14] Tello A Gatinel D Galvis V Prada AM Duarte LM Villamizar SJ. Importance of the appropriate history-taking process in patients with keratoconus to reduce the risk of finding spurious associations. Cornea. (2023). doi: 10.1097/ICO.0000000000003244 [Epub ahead of print].

[15] Mazharian A Panthier C Courtin R Jung C Rampat R Saad A. Incorrect sleeping position and eye rubbing in patients with unilateral or highly asymmetric keratoconus: a case-control study. Graefes Arch Clin Exp Ophthalmol Albrecht Von Graefes Arch Klin Exp Ophthalmol. (2020) 258:2431–9. doi: 10.1007/s00417-020-04771-z, PMID: 32524239 PMC7584543

[16] Hashemi H Heydarian S Hooshmand E Saatchi M Yekta A Aghamirsalim M. The prevalence and risk factors for keratoconus: a systematic review and Meta-analysis. Cornea févr. (2020) 39:263–10. doi: 10.1097/ICO.0000000000002150, PMID: 31498247

[17] Guilbert E Saad A Elluard M Grise-Dulac A Rouger H Gatinel D. Repeatability of keratometry measurements obtained with three topographers in Keratoconic and Normal corneas. J Refract Surg Thorofare NJ 1995. (2016) 32:187–2. doi: 10.3928/1081597X-20160113-01 27027626

[18] Gomes JAP Tan D Rapuano CJ Belin MW Ambrósio R Guell JL. Global consensus on keratoconus and ectatic diseases. Cornea. avr. (2015) 34:359–9. doi: 10.1097/ICO.0000000000000408 25738235

[19] Kandarakis A Karampelas M Soumplis V Panos C Makris N Kandarakis S. A case of bilateral self-induced keratoconus in a patient with tourette syndrome associated with compulsive eye rubbing: case report. BMC Ophthalmol. (2011) 11:28. doi: 10.1186/1471-2415-11-28 21936935 PMC3191478

[20] Wittig-Silva C Chan E Islam FMA Wu T Whiting M Snibson GR. A randomized, controlled trial of corneal collagen cross-linking in progressive keratoconus: three-year results. Ophthalmology Avr. (2014) 121:812–1. doi: 10.1016/j.ophtha.2013.10.028 24393351

[21] Schröder S Langenbucher A Schrecker J. Comparison of corneal elevation and pachymetry measurements made by two state of the art corneal tomographers with different measurement principles. PLoS One. (2019) 14:e0223770. doi: 10.1371/journal.pone.0223770, PMID: 31618270 PMC6795467

[22] McAlinden C Schwiegerling J Khadka J Pesudovs K. Corneal aberrations measured with a high-resolution Scheimpflug tomographer: repeatability and reproducibility. J Cataract Refract Surg avr. (2020) 46:581–10. doi: 10.1097/j.jcrs.0000000000000084, PMID: 32050208

[23] Defeat Keratoconus (2023) The keratoconus diary [internet]. Defeat keratoconus. [cité 17 mars 2023]. Disponible sur Available at: https://defeatkeratoconus.com/ (Accessed March 17, 2023).

[24] Gordon-Shaag A Millodot M Kaiserman I Sela T Barnett Itzhaki G Zerbib Y. Risk factors for keratoconus in Israel: a case-control study. Ophthalmic Physiol Opt J Br Coll Ophthalmic Opt Optom. (2015) 35:673–1. doi: 10.1111/opo.12237, PMID: 26286678

[25] Naderan M Shoar S Rezagholizadeh F Zolfaghari M Naderan M. Characteristics and associations of keratoconus patients. Contact Lens Anterior Eye J Br Contact Lens Assoc juin. (2015) 38:199–5. doi: 10.1016/j.clae.2015.01.008 25707930

[26] Salman A Darwish T Ghabra M Kailani O Haddeh Y Askar M. Prevalence of keratoconus in a population-based study in Syria. J Ophthalmol. (2022) 2022:1–9. doi: 10.1155/2022/6064533 PMC9246644 35783343

[27] Santodomingo-Rubido J Carracedo G Suzaki A Villa-Collar C Vincent SJ Wolffsohn JS. Keratoconus: an updated review. Contact Lens Anterior Eye J Br Contact Lens Assoc. juin. (2022) 45:101559. doi: 10.1016/j.clae.2021.101559, PMID: 34991971

[28] Mou Y Qin Q Huang X Jin X. Risk factors and severity of keratoconus on the East Coast of China. Int Ophthalmol juill. (2022) 42:2133–40. doi: 10.1007/s10792-022-02212-w, PMID: 35038123

[29] Song M Fang QY Seth I Baird PN Daniell MD Sahebjada S. Non-genetic risk factors for keratoconus. Clin Exp Optom. (2022):1–11. doi: 10.1007/978-981-19-4262-4_2 [Epub ahead of print]. 35504720

[30] Coyle JT. Keratoconus and eye rubbing. Am J Ophthalmol avr. (1984) 97:527–8. doi: 10.1016/S0002-9394(14)76143-4 6720826

[31] Gunes A Tok L Tok Ö Seyrek L. The youngest patient with bilateral keratoconus secondary to chronic persistent eye rubbing. Semin Ophthalmol. (2015) 30:454–6. doi: 10.3109/08820538.2013.874480, PMID: 24506444

[32] Fakhraie G Vahedian Z. Post filtering surgery globe massage-induced keratoconus in an eye with iridocorneal endothelial syndrome: a case report and literature brief review. J Ophthalmic Vis Res. (2016) 11:319–2. doi: 10.4103/2008-322X.158896, PMID: 27621792 PMC5000537

[33] Lindsay RG Bruce AS Gutteridge IF. Keratoconus associated with continual eye rubbing due to punctal agenesis. Cornea. juill. (2000) 19:567–9. doi: 10.1097/00003226-200007000-00034 10928781

[34] Bawazeer AM Hodge WG Lorimer B. Atopy and keratoconus: a multivariate analysis. Br J Ophthalmol août. (2000) 84:834–6. doi: 10.1136/bjo.84.8.834 PMC1723585 10906086

[35] Rabinowitz YS Galvis V Tello A Rueda D García JD. Genetics vs chronic corneal mechanical trauma in the etiology of keratoconus. Exp Eye Res janv. (2021) 202:108328. doi: 10.1016/j.exer.2020.108328, PMID: 33172608

[36] Ferdi AC Nguyen V Gore DM Allan BD Rozema JJ Watson SL. Keratoconus natural progression: a systematic review and Meta-analysis of 11 529 eyes. Ophthalmology juill. (2019) 126:935–5. doi: 10.1016/j.ophtha.2019.02.029 30858022

[37] Nath S Shen C Koziarz A Banfield L Nowrouzi-Kia B Fava MA. Transepithelial versus epithelium-off corneal collagen cross-linking for corneal ectasia: a systematic review and Meta-analysis. Ophthalmology août. (2021) 128:1150–60. doi: 10.1016/j.ophtha.2020.12.023, PMID: 33383093

[38] McAnena L Frank A Michael O’Keefe A. Cross-linking in children with keratoconus: a systematic review and meta-analysis. Acta Ophthalmol. (2017) 95:229–39. doi: 10.1111/aos.13224 27678078

[39] Or L Rozenberg A Abulafia A Avni I Zadok D. Corneal cross-linking in Pediatric patients: evaluating treated and untreated Eyes-5-year follow-up results. Cornea août. (2018) 37:1013–7. doi: 10.1097/ICO.0000000000001629, PMID: 29746325

[40] Achiron A El-Hadad O Leadbetter D Hecht I Hamiel U Avadhanam V. Progression of Pediatric keratoconus after corneal cross-linking: a systematic review and pooled analysis. Cornea. (2022) 41:874–8. doi: 10.1097/ICO.0000000000002808, PMID: 34294638

[41] Péterszegi G Robert AM Robert L Renard G. The importance of the Maillard reaction in ophtalmology. J Soc Biol. (2007) 201:209–4. doi: 10.1051/jbio:2007026, PMID: 17978755

[42] Perone JM Conart JB Bertaux PJ Sujet-Perone N Ouamara N Sot M. Mechanical Modeling of a Keratoconic cornea. Cornea. (2017) 36:1263–6. doi: 10.1097/ICO.0000000000001293, PMID: 28749895

[43] McMonnies CW Korb DR Blackie CA. The role of heat in rubbing and massage-related corneal deformation. Contact Lens Anterior Eye J Br Contact Lens Assoc août. (2012) 35:148–4. doi: 10.1016/j.clae.2012.01.001, PMID: 22309634

[44] McMonnies CW. The possible significance of the baropathic nature of keratectasias. Clin Exp Optom mars. (2013) 96:197–10. doi: 10.1111/j.1444-0938.2012.00726.x, PMID: 23614145

[45] McMonnies CW Boneham GC. Corneal responses to intraocular pressure elevations in keratoconus. Cornea. juill. (2010) 29:764–10. doi: 10.1097/ICO.0b013e3181ca2b75 20489581

[46] Osuagwu UL Alanazi SA. Eye rubbing-induced changes in intraocular pressure and corneal thickness measured at five locations, in subjects with ocular allergy. Int J Ophthalmol. (2015) 8:81–8. doi: 10.3980/j.issn.2222-3959.2015.01.15, PMID: 25709913 PMC4325247

[47] McMonnies CW. The evidentiary significance of case reports: eye rubbing and keratoconus. Optom Vis Sci Off Publ Am Acad Optom avr. (2008) 85:262–9. doi: 10.1097/OPX.0b013e318169287a, PMID: 18382341

[48] Ioannidis AS Speedwell L Nischal KK. Unilateral keratoconus in a child with chronic and persistent eye rubbing. Am J Ophthalmol févr. (2005) 139:356–7. doi: 10.1016/j.ajo.2004.07.044 15734005

[49] Bral N Termote K. Unilateral keratoconus after chronic eye rubbing by the nondominant hand. Case Rep Ophthalmol. (2017) 8:558–1. doi: 10.1159/000484712, PMID: 29422858 PMC5803714

[50] Saad A Rizk M Gatinel D. Fourteen years follow-up of a stable unilateral keratoconus: unique case report of clinical, tomographical and biomechanical stability. BMC Ophthalmol. (2022) 22:245. doi: 10.1186/s12886-022-02412-z 35658844 PMC9164538

[51] Millodot M Shneor E Albou S Atlani E Gordon-Shaag A. Prevalence and associated factors of keratoconus in Jerusalem: a cross-sectional study. Ophthalmic Epidemiol avr. (2011) 18:91–7. doi: 10.3109/09286586.2011.560747, PMID: 21401417

[52] Waked N Fayad AM Fadlallah A El Rami H. Keratoconus screening in a Lebanese students’ population. J Fr Ophtalmol. (2012) 35:23–9. doi: 10.1016/j.jfo.2011.03.016 21715046

[53] Assiri AA Yousuf BI Quantock AJ Murphy PJ. Incidence and severity of keratoconus in Asir province, Saudi Arabia. Br J Ophthalmol. (2005) 89:1403–6. doi: 10.1136/bjo.2005.074955, PMID: 16234439 PMC1772915

[54] Mazzotta C Traversi C Mellace P Bagaglia SA Zuccarini S Mencucci R. Keratoconus progression in patients with allergy and elevated surface matrix metalloproteinase 9 point-of-care test. Eye Contact Lens. (2018) 44:S48–53. doi: 10.1097/ICL.0000000000000432, PMID: 28991055

[55] Edwards M McGhee CN Dean S. The genetics of keratoconus. Clin Exp Ophthalmol. (2001) 29:345–1. doi: 10.1046/j.1442-9071.2001.d01-16.x 11778802

[56] McMahon TT Shin JA Newlin A Edrington TB Sugar J Zadnik K. Discordance for keratoconus in two pairs of monozygotic twins. Cornea. juill. (1999) 18:444–1. doi: 10.1097/00003226-199907000-00010, PMID: 10422858

[57] Goldich Y Marcovich AL Barkana Y Mandel Y Hirsh A Morad Y. Clinical and corneal biomechanical changes after collagen cross-linking with riboflavin and UV irradiation in patients with progressive keratoconus: results after 2 years of follow-up. Cornea. juin. (2012) 31:609–4. doi: 10.1097/ICO.0b013e318226bf4a 22378112

[58] Epstein RL Chiu YL Epstein GL. Pentacam HR criteria for curvature change in keratoconus and postoperative LASIK ectasia. J Refract Surg Thorofare NJ. (1995) 28:890–4. 10.3928/1081597X-20121115-04 23231740

[59] Maeda A. Can not touching the nose or eyes help cold prevention? Possibility of application using a smartwatch and self-checking. Annu Int Conf IEEE Eng Med Biol Soc IEEE Eng Med Biol Soc Annu Int Conf. (2020) 2020:5722–8. doi: 10.1109/EMBC44109.2020.9176589 33019274

[60] Vinciguerra P Albé E Frueh BE Trazza S Epstein D. Two-year corneal cross-linking results in patients younger than 18 years with documented progressive keratoconus. Am J Ophthalmol sept. (2012) 154:520–6. doi: 10.1016/j.ajo.2012.03.020, PMID: 22633357

